# Distribution of Class 1–3 Integrons in Carbapenem-Resistant *Pseudomonas aeruginosa* Isolated from Inpatients in Shiraz, South of Iran

**DOI:** 10.4314/ejhs.v31i4.5

**Published:** 2021-07

**Authors:** Malekzadegan Yalda, Tabatabaei Zahra Sadat, Rastegar Moghaddam Nosrati Elham, Tabatabaei Seyed Mohammad, Mohagheghzadeh Neda, Motamedifar Mohammad

**Affiliations:** 1 Department of Bacteriology and Virology, School of Medicine, Shiraz University of Medical Sciences, Shiraz, Iran; 2 Shiraz HIV/AIDS Research Center, Institute of Health, Shiraz University of Medical Sciences, Shiraz, Iran; 3 Student research Committee, School of Medicine, Shiraz University of Medical Sciences, Shiraz, Iran; 4 Depatment of Pathobiology, Faculty of Veterinary Medicine, Shiraz University, Shiraz, Iran

**Keywords:** Nosocomial infection, Carbapenem-resistant Pseudomonas aeruginosa, Integron

## Abstract

**Background:**

Health-care-associated infection (HAI) is effect on patients for the time of staying in the hospital. Opportunistic pathogens including Pseudomonas aeruginosa are the most dangerous biological agents in nosocomial infections. This study aimed to assess the prevalence of 3 classes of integrons carrying to carbapenem resistance in P. aeruginosa strains collected from Nemazee hospital.

**Methods:**

This cross-sectional study was conducted on clinical P. aeruginosa isolates were collected from Nemazee hospital. The identification of the isolates was performed by routine biochemical tests. Antimicrobial sensitivity testing was determined using the disk diffusion method against imipenem and meropenem. The int1, int2 and int3 genes were detected using the polymerase chain reaction (PCR).

**Results:**

Seventy-five clinical isolates of P. aeruginosa were recovered from various clinical infections. A carbapenem-resistant phenotype was detected in 42.7% (imipenem) and 29.3% (meropenem) of isolates. As the PCR results, 48 (64%) and 15 (20%) isolates were identified as being positive for class 1 and class 2 integrons, respectively. Class 3 integrons were not found among the studied isolates.

**Conclusions:**

Our data demonstrate the importance of class 1 and 2 integrons in carbapenem resistant P. aeruginosa strains. Therefore, integrons play an important role in acquisition and dissemination of carbapenem resistance genes among these pathogens, so, management of infection control policies and the appropriate use of antibiotics is essential for control the spreading of antibiotics resistance genes.

## Introduction

Healthcare associated infections (HAI), or nosocomial infections are known as infectious diseases that affect patients for the time of staying in the hospital and their manifestation apparent after 48–72 hours of hospitalization (1). Significantly, nosocomial infection is related to community health and is a risk factor for the spread of infections and directly related to the economy (2). In general, surgical procedures and invasive medical devices cause this type of infection(3). Opportunistic pathogens are the most dangerous biological agents in nosocomial infections (4). A range of multidrug-resistant organisms, including *Pseudomonas aeruginosa, Acinetobacter baumannii*, and extended-spectrum β-lactamase (ESBL) and carbapenemase-producing Enterobacteriaceae are the cause of nosocomial infections (5). Among them, *P. aeruginosa* is the major cause of nosocomial infections (6).

*P. aeruginosa* is a non-fermentative, aerobic, and rod-shaped bacterium that has minimal nutrition requirements (7). This organism is capable of causing severe infections such as sepsis, pneumonia, urinary tract infection, bacteremia and, soft-tissue infection (8). *P. aeruginosa* can cause infections in immunocompromised patients, including burn wounds, and cystic fibrosis (9). Infections caused by this bacteria are frequently problematic and life-threatening, therefore mainly correlated with high mortality and morbidity rates (10). The antimicrobial resistance in *P. aeruginosa* is one of the major public health concerns in the world. Imipenem and meropenem as carbapenems are the most effective antibiotics for the treatment of infections caused by (multi-drug resistant) MDR strains of *P. aeruginosa*, but intensive clinical use of carbapenems has caused an increase in carbapenem resistance (11). Integrons are potentially mobile genetic elements that capture and incorporate gene cassettes by sites specific recombination and convert them to functional genes. They play a central role in the dissemination of resistance genes among gram-negative bacteria, especially in *Pseudomonas* (12,13). This element contains three necessary components including the *int*I encoding an integrase gene, *att*I encoding the attachment site and the promoter region (PC) (14). Today, in Gram-negative bacteria, three types of integrons have been recognized with different *int* genes (IntI1, IntI2, and IntI3), that are associated with resistance gene cassettes (14). However, class 1 integron is the most widespread among clinical isolates of *P. aeruginosa*, which confer resistance to aminoglycosides, beta-lactams, chloramphenicol, carbapenems, macrolides agents (15).

Evidence recommends that integrons are coding and responsible for the dissemination of carbapenem resistance among *P. aeruginosa* isolates (16). The results of this study can be relatively important for choosing an effective antibiotic to manage infection of *P. aeruginosa* in the hospital environment. This study aimed to assess the prevalence of 3 classes of integrons in carbapenem-resistant *P. aeruginosa* strains collected from Nemazee hospital.

## Materials and Methods

**Bacterial isolates and identification**: Seventy-five non-duplicated clinical *P. aeruginosa* isolates were recovered from various clinical infections to Nemazee hospital from August 2015 to February 2016. These strains were isolated from various clinical infections including urinary tract infection (UTI), respiratory tract infection (RTI), eye infection (EI), bloodstream infection (BSI), skin and soft tissue infection (SSTI), and abdominal infection (AI). The identification of the intended isolates was performed by using routine biochemical tests as previously described (17).

**Antibiotic susceptibility testing**: Antimicrobial sensitivity testing was carried out using the disk diffusion method on Muller-Hinton agar against imipenem and meropenem as previously recommended in the Clinical and Laboratory Standards Institute (CLSI) (18). All antibiotic disks were provided from Mast Co., UK. The plates were incubated for 24 h at 37°C. Then, the results were interpreted as susceptible, intermediately susceptible and resistant. Intermediate-susceptible isolates were considered as resistant. *P. aeruginosa* ATCC27853 was included as the control strain in each run of drug susceptibility testing.

**Detection of *int* genes by PCR**: Deoxyribonucleic acid (DNA) extraction for the detection of integron genes was performed by the boiling method as previously described (19). The extracted DNA was stored at -20°C for its use as a DNA template in PCR reactions. The detection of *int1, int2 and int3* genes were carried out using the polymerase chain reaction (PCR) by the set of primers are listed in Table 1. PCR mix was performed in a final volume of 25 µl containing 2 µL template DNA, 0.2 mM of each deoxynucleoside triphosphate, 10 pmol of each primer, 10 mM Tris-HCl, 1.5 mM MgCl2, 50 mMKCl, and 1.5 U of Taq DNA polymerase. The amplification was achieved in a T100™ thermal cycler (Bio-Radd, Hercules, CA, USA), with the following program: initial denaturation at 94°C for 5 min, 30 cycles of denaturation at 94°C for 60s, primer annealing at 57°C for 60s, extension at 72°C for 2 min and a final extension at 72°C for 10 min. The PCR products were separated using 1.5% agarose gel electrophoresis containing safe DNA stain and visualized in the UV illuminator.

**Table 1:** Primers to *int* genes utilized in PCR

Primer	Sequence (5′ to 3′)	Amplicon Size (bp)	References
*int1*-F	GGTCAAGGATCTGGATTTCG	483	20
*int1*-R	ACATGCGTGTAAATCATCGTC		
*int2*-F	CACGGATATGCGACAAAAAGGT	789	20
*int2*-R	GTAGCAAACGAGTGACGAAATG		
*int3*-F	AGTGGGTGGCGAATGAGTG	600	20
*int3*-R	TGTTCTTGTATCGGCAGGTG		

**Data analysis**: Analysis was performed by using SPSSTM software, version 21.0 (IBM Corp., USA). The results are presented as descriptive statistics in terms of relative frequency. Values were expressed as the mean ± standard deviation (continuous variables) or percentages of the group (categorical variables).

## Results

In this cross-sectional study, 75 clinical isolates of *P. aeruginosa* were examined. Forty and thirty-five of isolates were from male and female patients, respectively. The range of age among patients was from 10 days to 77 years-old. All isolates of *P. aeruginosa* were recovered from different clinical infections including 26 (34.6%) isolates from urinary tract infection, 25 (33.3%) isolates from respiratory tract infection, nine (12%) isolates from bloodstream infection, five (6%) isolates from each eye, skin and soft tissue and abdominal infections. Intensive care unit with 39 isolates (52%), and transplant unit with 5 isolates (6%), were comprised of the highest and lowest rates of *P. aeruginosa* isolation respectively.

A carbapenem-resistant phenotype was detected in 42.7% (imipenem) and 29.3% (meropenem) of *P. aeruginosa* isolates. As the PCR results, 48 (64%) and 15 (20%) isolates were identified as being positive for class 1 and class 2 integrons, respectively. Class 3 integrons were not found among the studied isolates. Frequency of class 1 and class 2 integrons were 29 (60%) and 8 (53.3%) in imipenem resistance isolates, respectively. Whereas in meropenem resistance isolates these frequencies were 19 (86%) and 7 (31.8%), respectively. 7 (21.8%) imipenem resistant isolates and 6 (27.2%) meropenem resistant isolates harbored both integrons (class 1 and 2).

The distribution of class 1 integron among the isolates according to originated clinical infections was highest for urine (46.2%), and respiratory tract (30.8%) infections (Table 2). As shown in Table 2, among the clinical infections, the frequency of class 2 integron is high (69.2%) in respiratory tract infections. The majority of isolates harbored class 1 and 2 integrons were from the ICU ward (48.7%, 46.1%) followed by Internal (33.3%, 38.5%), Surgery (12.8%, 7.7%) and Transplant (5.2%, 7.7%) wards.

**Table 2:** The frequency of class 1 and 2 integrons among *P. aeruginosa* isolates according to the type of infections

Infection	Class I integron No. (%)	Class II integron No. (%)
RTI	12 (30.8)	9 (69.2)
EI	2 (5.1)	1 (7.7)
BSI	4 (10.2)	0
UTI	18 (46.2)	3 (23.1)
SSTI	1 (2.6)	0
AI	2 (5.1)	0

UTI: urinary tract infection, RTI: respiratory tract infection, EI: eye infection, BSI: bloodstream infection, SSTI: skin and soft tissue infection, AI: abdominal infection

## Discussion

There are restricted therapeutic choices for the treatment of *P. aeruginosa.* Therefore treatment by the last line of drugs is considered for the management of infections created by these bacteria (20). A recent study have revealed that many resistance genes can be transferred by integrons (21). Integron particularly contributes to the horizontal transmission of genes involved in antibiotic resistance among clinical isolates of *P. aeruginosa* (22). This study aimed to investigate the presence of class 1, 2 and 3 integrons in *P. aeruginosa* isolated from southwest of Iran.

In the present study, the resistance rate to carbapenem in *P. aeruginosa* isolates was 42.6%. This rate was reported ranging 11% to 61% in Iranian studies (23–26), while other countries showed ranging from 12.9% to 38% (27–31). Several factors are involved in these differences between the regions such as the variation in the studied population, infection control policy, and testing methods. Also, it has been suggested that three factors are significantly associated with the emergence of carbapenem-resistant *P. aeruginosa* isolates including MDR phenotype, hospitalization in burn units and the presence of class 1 integrons (22).

Although it is clear that multiple mechanisms are related to antibiotic resistance in *P. aeruginosa* isolates, the role of integrons in the resistance acquisition of this bacterium are inevitable (32). The integrons screening in this study reveals that the majority of (64%) *P. aeruginosa* isolates carried class 1 integrons. Numerous studies on different clinical samples reported the prevalence of integrons among *P. aeruginosa* isolates from Iran and other parts of the world, and most of them reported a higher incidence of class 1 integron (16, 20, 33–36). Also, the prevalence rate of class 1 integrons detected in this study is higher than the previously reported rate of from Brazil (41.5%) (37), and China (45.8 %) (38). This low rate is possibly due to the difference in the geographical area and the studied population.

In line with our findings, several previous studies showed a low rate of class 2 integrons in clinical isolates of *P. aeruginosa* (21, 35, 36, 38–41). The absence or sporadic detection of class 2 integrons were confirmed the restricted distribution of these genetic elements among bacterial populations.

In summary, Our data demonstrate the importance of class 1 and 2 integrons in carbapenem resistant *P. aeruginosa* strains isolated from different parts of the hospital that suggest the high potential of this structure to be transferred among bacteria by the horizontal gene transfer apparatus. We reported a high prevalence of class 1 integrons in clinical isolates of *P. aeruginosa* whereas the frequency of class 2 integrons was low. This difference indicates a high risk of resistance transmission and dissemination of integron producing isolates in hospitals. Therefore, integrons play an important role in acquisition and dissemination of carbapenem resistance genes among these pathogens, so, management of infection control policies and the appropriate use of antibiotics is essential for control the spreading of antibiotics resistance genes.

The lack of investigation and characterization of resistance gene cassettes associated with class 1 and 2 integrons can be mentioned as one of the main limitations of the present study.
